# Supplementary material for: Colorectal Cancer Bioengineered Microtissues as a Model to Replicate Tumor-ECM Crosstalk and Assess Drug Delivery Systems In Vitro
Source: Int J Mol Sci. 2023 Mar 16;24(6):5678. doi: 10.3390/ijms24065678 (PMC10059762; doi:10.3390/ijms24065678)
Supplement: Supplementary file 1 [file ijms-24-05678-s001.zip › ijms-2224451-supplementary.pdf]

International Journal of *Molecular Sciences*

Supplementary figures

Colorectal cancer bioengineered microtissues as model to replicate tumor-ECM cross-talk and assess drug delivery systems in vitro

Alessia La Rocca [1,2], Vincenza De Gregorio [3,4], Giorgia Imparato [1,3,5,*], Elena Lagreca [1,5], Raffaele Vecchione [1], Paolo Antonio Netti [1,3,5]

[1] Center for Advanced Biomaterials for Health Care (CABHC), Istituto Italiano di Tecnologia, 80125 Napoli, Italy
[2] San Raffaele Diabetes Research Institute, IRCCS San Raffaele Scientific Institute, 20132 Milan, Italy
[3] Interdisciplinary Research Centre on Biomaterials (CRIB), University of Naples Federico II, 80125 Naples, Italy
[4] Department of Biology, University of Naples Federico II, Complesso Universitario Monte S.Angelo, 80126 Naples, Italy
[5] Department of Chemical Materials and Industrial Production (DICMaPI), University of Naples Federico II, 80125 Naples, Italy
* Correspondence: giorgia.imparato@iit.it (G.I.);

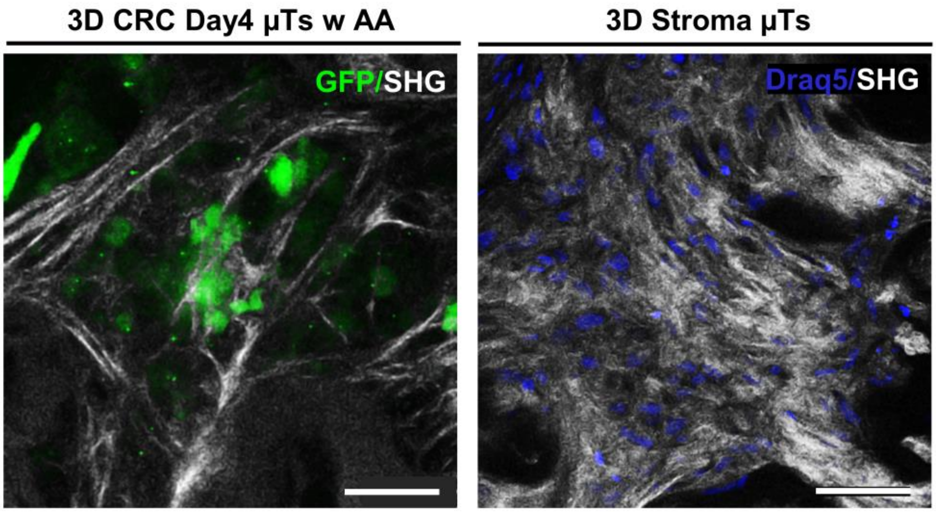

Figure S1. High magnification of different organization and structure of auto-produced ECM in 3D CRC Day4 µTs and 3D Stroma µTs; scale bar 40 µm.

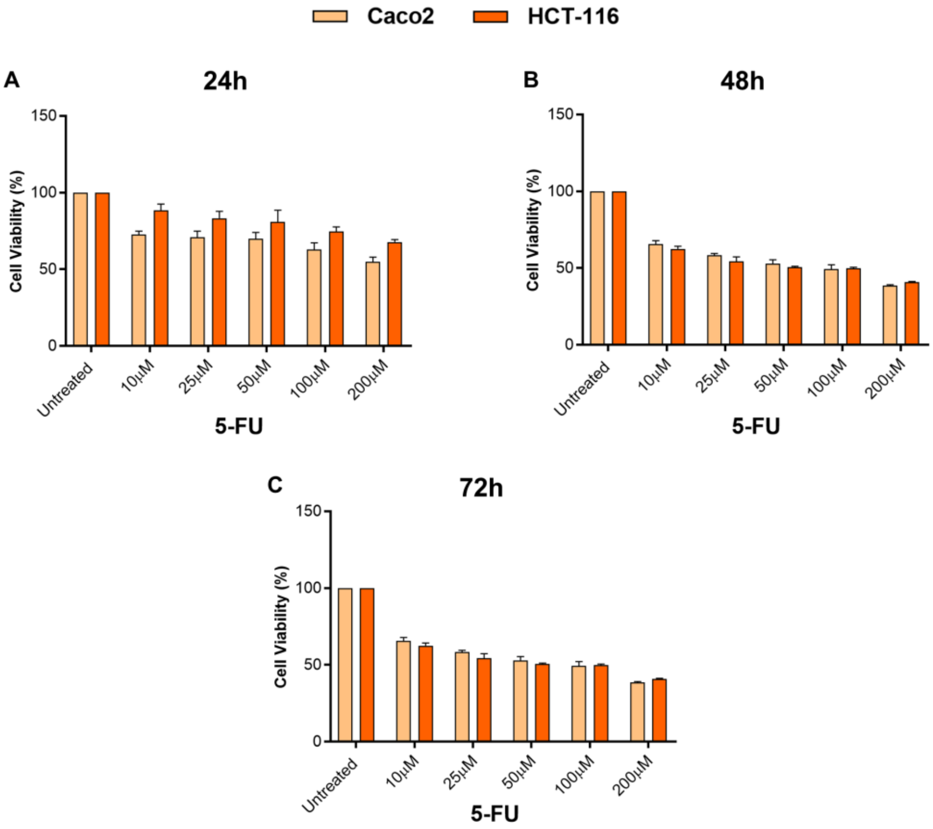

Figure S2. Absence of selective cytotoxic effect of 5-FU between less invasive colon cancer cells (Caco2) and more invasive cancer cells (HCT-116) in 2D *in vitro* cell cultures. Caco2 and HCT-116 were treated with different 5-FU concentrations and MTT analyses were carried out after 24 h, 48 h and 72 h of treatments. All the experiments were performed in triplicate (n = 3), values represent the mean and mean-standard error.

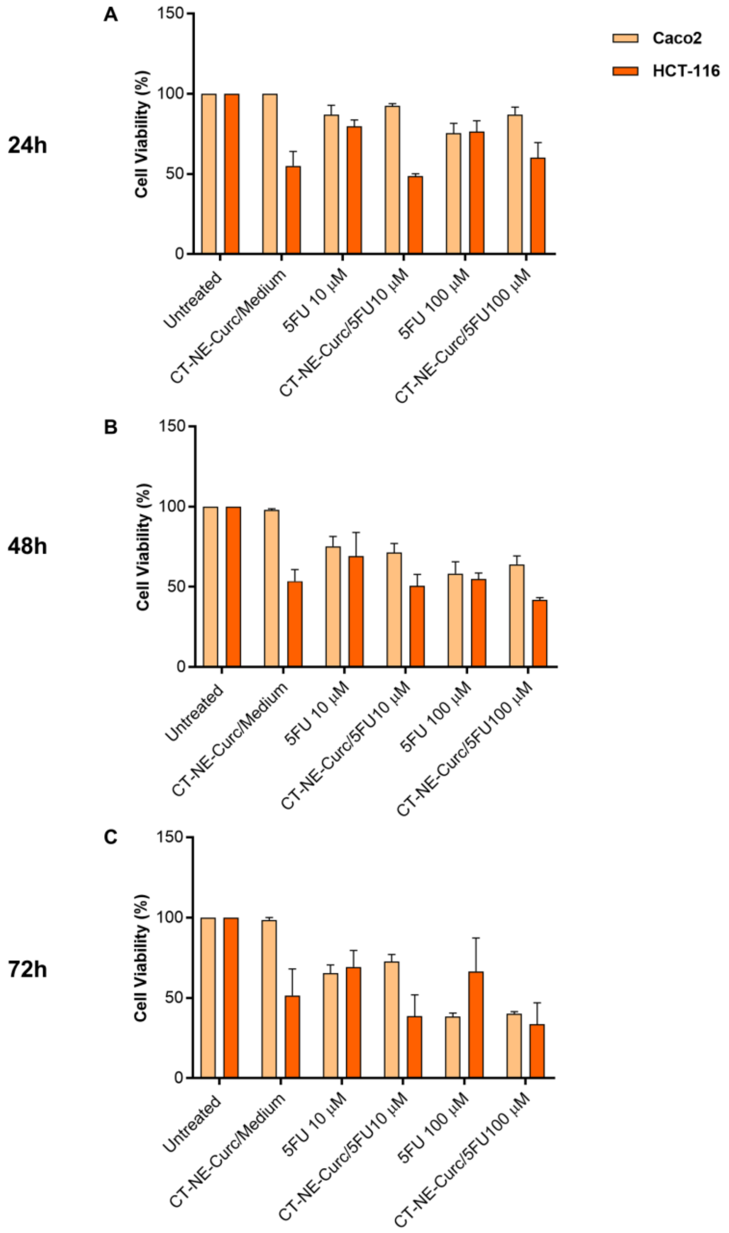

Figure S3. Synergistic effects of CT-NE-Curc and 5-FU 10 µM and 100 µM in 2D *in vitro* colon cancer cell cultures. Caco2 and HCT-116 cells were pre-treated with CT-NE-Curc for 2 h and then treated with 5-FU 10 µM and 100 µM. MTT analyses were carried out after 24 h, 48 h and 72 h of treatments. All the experiments were performed in triplicate (n = 3), values represent the mean and mean-standard error.

1

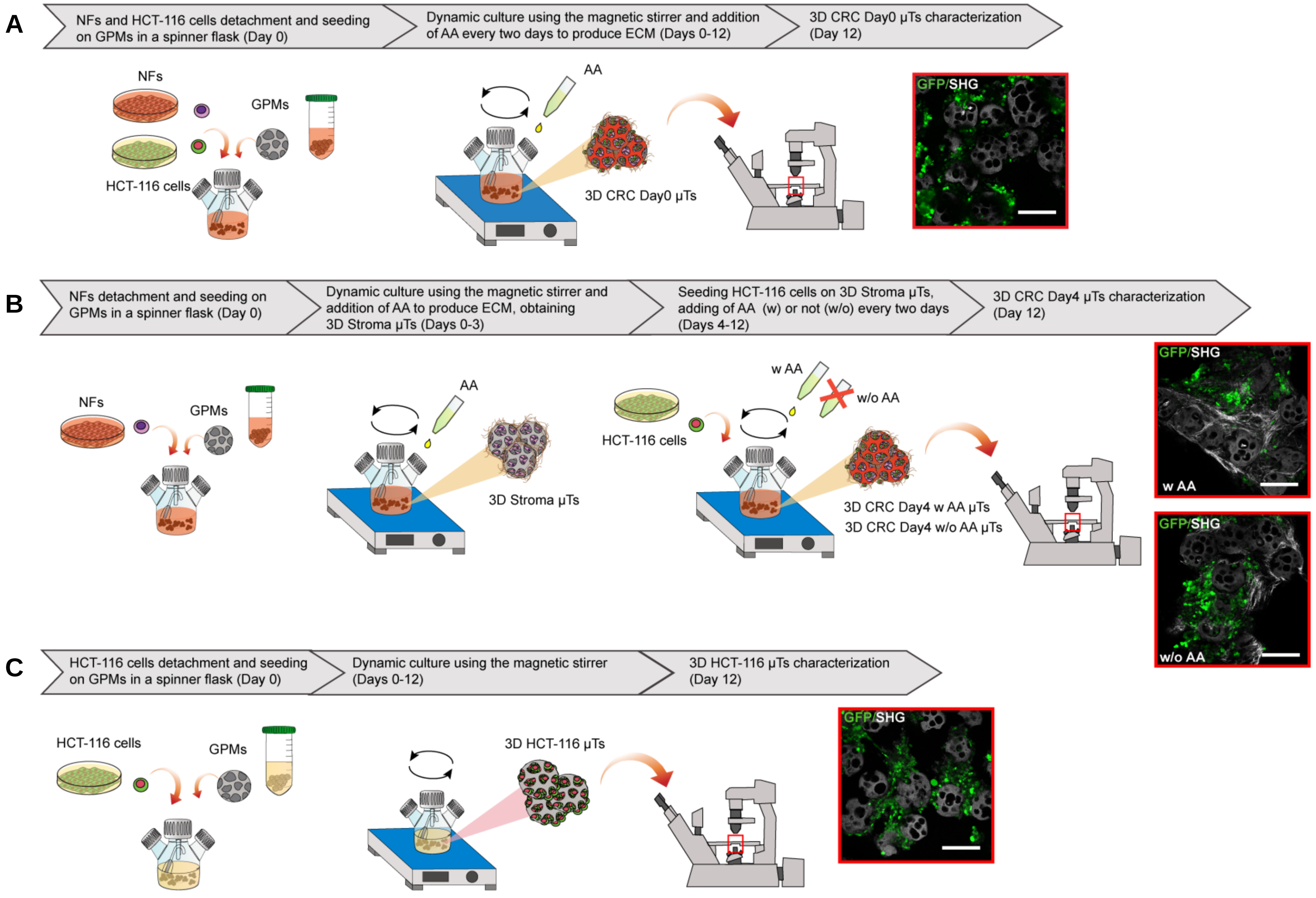
A
NFs and HCT-116 cells detachment and seeding on GPMs in a spinner flask (Day 0)
Dynamic culture using the magnetic stirrer and addition of AA every two days to produce ECM (Days 0-12)
3D CRC Day0 μTs characterization (Day 12)
NFs
GPMs
HCT-116 cells
AA
3D CRC Day0 μTs
GFP/SHG
B
NFs detachment and seeding on GPMs in a spinner flask (Day 0)
Dynamic culture using the magnetic stirrer and addition of AA to produce ECM, obtaining 3D Stroma μTs (Days 0-3)
Seeding HCT-116 cells on 3D Stroma μTs, adding of AA (w) or not (w/o) every two days (Days 4-12)
3D CRC Day4 μTs characterization (Day 12)
NFs
GPMs
AA
3D Stroma μTs
HCT-116 cells
w AA
w/o AA
3D CRC Day4 w AA μTs
3D CRC Day4 w/o AA μTs
GFP/SHG
w AA
GFP/SHG
w/o AA
C
HCT-116 cells detachment and seeding on GPMs in a spinner flask (Day 0)
Dynamic culture using the magnetic stirrer (Days 0-12)
3D HCT-116 μTs characterization (Day 12)
HCT-116 cells
GPMs
3D HCT-116 μTs
GFP/SHG

International Journal of *Molecular Sciences*

MDPI

Figure S4: A schematic diagram of the sequence of steps for 3D µTs fabrication. (A) 3D CRC Day0 µTs production; (B) 3D Stroma µTs and 3D CRC Day 4 w or w/o µTs production; (C) 3D HCT-116 µTs production. Normal fibroblasts (NFs); Gelatin porous microbeads (GPMs); Ascorbic Acid (AA); 3D CRC Day4 µTs with Ascorbic Acid (w A) and 3D CRC Day4 without Ascorbic acid (w/o AA); Green Fluorescent Protein (GFP); Second Harmonic Generation (SHG).
